# From IL-6 production to IL-6R buffering: a unified IL-6-IL-6R framework for immune homeostasis during viral infection

**DOI:** 10.3389/fimmu.2026.1955147

**Published:** 2026-09-22

**Authors:** Ashraf S. Yousif

**Affiliations:** Department of Infectious Disease Research, King Abdullah International Medical Research Center, King Saud bin Abdulaziz University for Health Sciences, Ministry of National Guard Health Affairs, Riyadh, Saudi Arabia

**Keywords:** cytokine buffering, dengue virus (DENV), IL6 rs1800795, IL6 rs2069843, IL6R rs2228145, immune homeostasis, interleukin-6 (IL-6), soluble IL-6 receptor (sIL-6R)

## Abstract

Interleukin-6 (IL-6) is a pleiotropic cytokine that coordinates innate and adaptive immunity and maintains tissue homeostasis during infection and inflammation. Although transient IL-6 production is essential for host defense, dysregulated or sustained IL-6 activity contributes to immunopathology across infectious and inflammatory diseases. Dengue virus (DENV) infection provides an important context in which this balance may become disrupted. Elevated circulating IL-6 levels are associated with endothelial dysfunction, vascular leakage, coagulopathy, and severe dengue; however, the mechanisms regulating IL-6 bioavailability, signaling duration, and resolution remain incompletely understood. Genetic variation within *IL6* and *IL6R* may influence distinct but interconnected layers of IL-6 regulation. The intronic *IL6* rs2069843 and promoter rs1800795 variants have been associated with dengue susceptibility and severity in some populations and proposed to influence IL-6 expression, although their effects are not consistently replicated. In contrast, the functional *IL6R* rs2228145 (Asp358Ala; also known as rs8192284) variant enhances shedding of membrane-bound IL-6R and increases circulating soluble IL-6R (sIL-6R), thereby altering the extracellular context of IL-6 signaling. Importantly, increased sIL-6R may either facilitate IL-6 trans-signaling or contribute to extracellular buffering depending on the availability of soluble gp130 (sgp130). Experimental evidence for a dendritic cell-derived sIL-6R buffering system provides a physiological framework for understanding this regulation during acute viral infection. In this review, we integrate evidence from human genetics, cytokine biology, receptor signaling, and dengue immunopathogenesis to propose a unified, hypothesis-driven framework of IL-6 regulation. We conceptualize IL6 variants as potential modifiers of IL-6 production, whereas *IL6R* rs2228145 may modify soluble receptor buffering capacity and the extracellular distribution of IL-6. We further hypothesize that the dynamic balance among IL-6 production, sIL-6R availability, and sgp130-mediated neutralization, together with receptor abundance and ligand-receptor kinetics, may determine the intensity and duration of IL-6 activity more effectively than circulating IL-6 concentration alone. This framework provides experimentally testable hypotheses for understanding inter-individual variation in IL-6 homeostasis and dengue disease progression and may inform future biomarker development and precision immunomodulatory strategies.

## Introduction

### Immune homeostasis and the IL-6 signaling axis

The ability of the immune system to rapidly eliminate invading pathogens while limiting collateral tissue damage is a defining feature of effective host defense. This delicate equilibrium, commonly referred to as immune homeostasis, depends on the precise temporal and spatial regulation of inflammatory mediators (1–3). Among these, cytokines act as critical molecular messengers that coordinate communication between innate and adaptive immune cells, initiate antimicrobial responses, promote tissue repair, and orchestrate the resolution of inflammation (4–7). While robust cytokine production is indispensable for controlling infection, excessive or prolonged cytokine signaling can disrupt immune homeostasis, resulting in endothelial injury, vascular dysfunction, coagulopathy, and multi-organ pathology (8–11). Consequently, the magnitude, duration, and resolution of cytokine responses are tightly regulated through multiple complementary mechanisms that collectively preserve host integrity (12–14).

Among the pleiotropic cytokines involved in the regulation of inflammation, interleukin-6 (IL-6) occupies a central position at the interface between protective immunity and immunopathology (15–17). Produced by diverse cell types, including dendritic cells, monocytes, macrophages, endothelial cells, fibroblasts, and epithelial cells, IL-6 is rapidly induced following the recognition of pathogen-associated molecular patterns by innate immune receptors. Through activation of the Janus kinase (JAK)-signal transducer and activator of transcription 3 (STAT3) pathway, together with MAPK and PI3K-AKT signaling cascades, IL-6 coordinates acute-phase responses, promotes B cell differentiation, supports T follicular helper cell development, influences T helper cell polarization, and regulates tissue repair (18–21). These diverse functions underscore its indispensable role in antimicrobial immunity, while simultaneously explaining why dysregulated IL-6 signaling contributes to numerous inflammatory, autoimmune, and infectious diseases (15, 22–25).

Traditionally, studies investigating IL-6 biology have predominantly focused on the mechanisms governing cytokine production, including transcriptional activation, inflammasome-independent induction, and upstream signaling through pattern-recognition receptors (26–30). Elevated circulating IL-6 concentrations have therefore been widely interpreted as a direct surrogate of inflammatory intensity (23, 31–33). However, increasing evidence indicates that cytokine concentration alone cannot fully explain the biological activity of IL-6 or the marked heterogeneity of clinical outcomes observed among individuals exposed to similar inflammatory stimuli. Instead, the biological effects of IL-6 are increasingly recognized to depend not only on the quantity of cytokine produced but also on its persistence, bioavailability, receptor dynamics, and tissue responsiveness (34–38).

Recent progress in cytokine biology has started to change this view. The discovery of a dendritic cell-derived soluble receptor buffering system indicates that circulating IL-6 is actively regulated post-secretion via physiological mechanisms that affect cytokine persistence and receptor availability. This emerging concept raises the possibility that immune homeostasis is determined not solely by cytokine production but also by endogenous buffering processes that modulate the duration and magnitude of downstream signaling (39). Such a framework provides a new lens through which host genetic variation affecting either cytokine production or receptor biology can be interpreted.

Dengue virus (DENV) infection offers a compelling clinical model for exploring these mechanisms. Severe dengue is consistently associated with markedly elevated IL-6 concentrations, endothelial activation, vascular leakage, and systemic hyperinflammation, yet substantial inter-individual variability exists in disease severity despite comparable viral exposure (15, 40–49). This observation suggests that host-specific regulatory mechanisms, including inherited variation within the IL-6–IL-6R signaling axis, contribute to determining whether IL-6 responses remain protective or progress toward pathological inflammation. In this review, we propose that genetic variation within IL-6 and IL-6R represents two complementary dimensions of cytokine regulation: IL-6 variants primarily influence the magnitude of IL-6 production, whereas IL-6R variants may modify cytokine persistence and bioavailability through soluble receptor dynamics. Together, these mechanisms form the basis of an integrated model of IL-6 homeostasis that provides a conceptual framework for understanding host susceptibility to severe dengue and, more broadly, the regulation of inflammatory responses during acute viral infection.

## IL-6 biology during viral infection

Interleukin-6 (IL-6) is a pleiotropic cytokine that occupies a central position in the orchestration of antiviral immunity (15, 16, 22, 23). IL-6 is not an antiviral cytokine; it is an immunological coordinator that brings together innate pathogen sensing with adaptive immune activation, tissue repair, and the restoration of homeostasis (22, 23, 50). During acute viral infection, transient IL-6 production is essential for mounting an effective host response by promoting leukocyte recruitment, inducing acute-phase proteins, enhancing antibody production, and supporting T-cell differentiation (4–6). However, failure to appropriately regulate IL-6 signaling transforms this protective mediator into a major driver of immunopathology (9, 10, 17, 40). Consequently, IL-6 exemplifies the fundamental principle that the biological consequences of cytokine signaling depend not only on the initiation of inflammatory responses but also on their magnitude, duration, and timely resolution.

## Induction of IL-6 following viral infection

Viral infection rapidly activates IL-6 production through the recognition of viral nucleic acids and structural proteins by host pattern-recognition receptors (PRRs). Endosomal Toll-like receptors (TLR3, TLR7, and TLR8) detect viral RNA, whereas cytosolic sensors, including RIG-I and MDA5, recognize replicating viral genomes. These pathways converge on transcription factors such as NF-κB, AP-1, and interferon regulatory factors (IRFs), which rapidly induce IL6 transcription (18–21). Activated monocytes, macrophages, dendritic cells, neutrophils, endothelial cells, epithelial cells, and fibroblasts subsequently release IL-6 into the circulation, where its concentration often reflects the intensity of innate immune activation.

The induction of IL-6 occurs early during infection and functions in concert with type I interferons, tumor necrosis factor-α (TNF-α), and interleukin-1β (IL-1β) to establish an antiviral state (15, 51, 52). In contrast to interferons, which primarily restrict viral replication, IL-6 coordinates communication between innate and adaptive immune compartments, thereby shaping the magnitude and quality of subsequent immune responses.

## IL-6 signaling pathways

The biological effects of IL-6 are mediated through two interconnected signaling pathways: classical signaling and trans-signaling (**Figure 1**). In classical signaling, IL-6 binds to the membrane-bound IL-6 receptor (mIL-6R, CD126), which is expressed on a restricted range of cells, including hepatocytes, neutrophils, monocytes, and selected lymphocyte populations. The IL-6/mIL-6R complex associates with the signal-transducing receptor glycoprotein 130 (gp130), initiating intracellular activation of Janus kinases (JAKs) and phosphorylation of signal transducer and activator of transcription 3 (STAT3). Additional activation of the MAPK and PI3K–AKT pathways further regulate cell proliferation, differentiation, metabolism, and survival. Classical IL-6 signaling is generally considered essential for physiological immune responses, tissue regeneration, and acute-phase protein synthesis (13, 53, 54).

**Figure 1:**
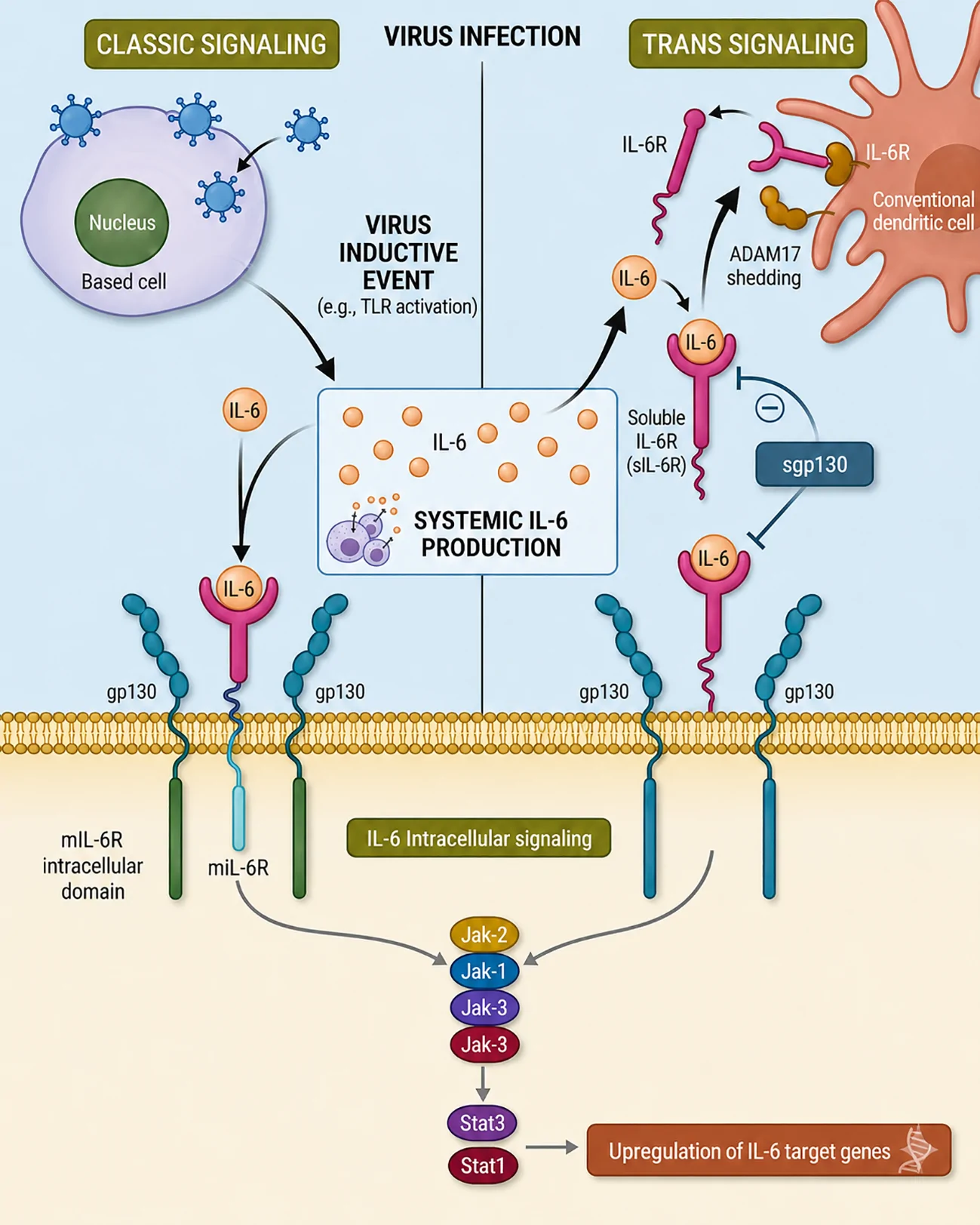
Mechanistic divergence of classic and trans-signaling pathways of interleukin-6 (IL-6) during viral infection.

In contrast, IL-6 trans-signaling expands the spectrum of IL-6-responsive cells. Proteolytic cleavage of membrane IL-6R by the metalloproteases ADAM10 and ADAM17 generates soluble IL-6 receptor (sIL-6R), which retains full ligand-binding capacity. The IL-6/sIL-6R complex subsequently binds membrane gp130 expressed on virtually all nucleated cells, enabling IL-6 signaling in tissues lacking membrane IL-6R expression, particularly vascular endothelial cells. This mechanism substantially broadens the inflammatory reach of IL-6 and has been implicated in chronic inflammation, vascular dysfunction, autoimmune disease, and severe viral infections (14, 17, 55). IL-6 trans-signaling is physiologically restrained by soluble gp130 (sgp130), which binds the IL-6/sIL-6R complex and forms an inactive IL-6/sIL-6R/sgp130 complex, thereby limiting access of the cytokine–receptor complex to membrane-bound gp130 and selectively suppressing trans-signaling (56).

## Biological functions of IL-6 during antiviral immunity

IL-6 exerts diverse immunological functions that extend across both innate and adaptive immunity. During the early phase of infection, IL-6 stimulates hepatic production of acute-phase proteins, including C-reactive protein (CRP), serum amyloid A, fibrinogen, and complement components, thereby enhancing pathogen recognition and clearance (50, 57, 58). Simultaneously, IL-6 promotes neutrophil mobilization from the bone marrow and facilitates recruitment of inflammatory leukocytes to infected tissues (59–61).

Within adaptive immunity, IL-6 influences multiple aspects of lymphocyte differentiation. Together with IL-21, IL-6 promotes differentiation of T follicular helper (Tfh) cells, supporting germinal center formation, affinity maturation, and long-lived antibody responses (62–64). In the presence of transforming growth factor-β (TGF-β), IL-6 drives differentiation of T helper 17 (Th17) cells while suppressing the generation of regulatory T cells, thereby amplifying inflammatory responses (65–67). IL-6 also contributes directly to plasma cell differentiation and sustained immunoglobulin production, highlighting its essential role in protective antiviral immunity (39, 68–70).

## Dysregulated IL-6 signaling and viral immunopathology

Although transient IL-6 signaling is indispensable for host defense, sustained activation contributes to tissue injury during numerous viral infections, including dengue, influenza, SARS-CoV-2, Ebola virus disease, and hantavirus infection (22, 23, 36, 40, 71–76). In severe dengue, endothelial activation and vascular leakage arise from a complex and coordinated inflammatory response involving multiple cytokines, vasoactive mediators, complement pathways, platelet dysfunction, and disruption of the endothelial glycocalyx. IL-6 may contribute to this process by promoting endothelial activation, increasing vascular permeability, inducing adhesion molecules such as ICAM-1 and VCAM-1, and stimulating VEGF production. However, these effects occur within a broader inflammatory network that includes TNF-α, IL-1β, IFN-γ, VEGF, complement activation, platelet dysfunction, and glycocalyx degradation. Persistent or dysregulated IL-6 signaling may therefore amplify and sustain endothelial and inflammatory responses rather than acting as an independent or primary driver of vascular leakage. The interplay among these pathways can enhance leukocyte recruitment, compromise vascular barrier integrity, activate coagulation and complement pathways, and ultimately contribute to the vascular dysfunction and systemic hyperinflammation characteristic of severe dengue. Thus, IL-6 should be considered an important component of the interconnected cytokine network that regulates disease severity, rather than a singular determinant of endothelial dysfunction or vascular leakage (17, 31, 45, 47, 60, 72, 76, 77).

Importantly, disease severity frequently correlates imperfectly with circulating IL-6 concentrations alone. Patients exhibiting similar plasma IL-6 levels often experience markedly different clinical outcomes, suggesting that cytokine concentration is not only one determinant of biological activity. Factors including receptor availability, soluble receptor dynamics, cytokine clearance, tissue responsiveness, and host genetic variation collectively influence the magnitude and duration of IL-6 signaling (34–38, 78–80).

## The missing dimension of IL-6 biology: from cytokine production to cytokine persistence

For more than three decades, investigations of IL-6 biology have focused predominantly on understanding how inflammatory stimuli regulate cytokine production. Extensive research has elucidated the molecular pathways controlling IL-6 transcription, including activation of pattern-recognition receptors, NF-κB, AP-1, and interferon regulatory factors, as well as numerous genetic polymorphisms that influence cytokine expression (29, 30, 81, 82). As a result, high levels of IL-6 in the blood are now widely accepted as both a biomarker and a cause of disease severity in infections, autoimmune disorders, and inflammatory syndromes (7, 8, 31, 32, 45, 72, 77, 82). This production-centric view has substantially advanced our understanding of IL-6 biology, yet it does not fully explain the remarkable variability in clinical outcomes observed among individuals with apparently similar inflammatory responses.

An important unresolved question is whether the quantity of IL-6 produced is the sole determinant of its biological activity. Clinical observations from dengue and other inflammatory diseases indicate that patients with comparable circulating IL-6 concentrations may experience markedly different degrees of tissue injury, vascular dysfunction, and systemic inflammation. These findings suggest that cytokine concentration alone cannot fully account for the intensity or duration of downstream signaling. Rather, the biological effects of IL-6 are likely to depend on additional regulatory processes that determine how long IL-6 remains biologically available, where it can signal, and how efficiently it engages its receptors (11, 34–38, 83, 84).

This perspective shifts attention from cytokine production to cytokine persistence as a complementary dimension of immune regulation. Following secretion, IL-6 enters a dynamic extracellular environment in which receptor binding, soluble receptor interactions, cytokine clearance, tissue distribution, and receptor-mediated signaling collectively influence its biological fate. These post-secretory processes are increasingly recognized as active regulators of cytokine activity rather than passive consequences of inflammation. Accordingly, immune homeostasis may be determined not only by the magnitude of cytokine production but also by the mechanisms that govern cytokine persistence, bioavailability, and signal duration (11, 19, 39).

Recent experimental findings support this broader view by demonstrating that soluble cytokine receptors can actively regulate circulating cytokine kinetics. Rather than functioning solely as signaling intermediates, soluble receptors may participate in physiological buffering systems that transiently sequester cytokines, modulate receptor accessibility, and influence the balance between cytokine preservation and biological activity (39). Building upon these observations, we propose that IL-6 homeostasis is governed by two complementary and interconnected regulatory dimensions. The first is cytokine production, which determines the magnitude of the initial inflammatory response and is influenced by genetic variation within IL-6, including rs2069843 and rs1800795 (-174 G>C) (81, 85–90). The second is cytokine persistence, which determines the duration and spatial distribution of IL-6 activity after secretion and may be modified by receptor biology, soluble receptor dynamics, and functional variation within IL-6R, particularly rs2228145 (Asp358Ala) (91).

This conceptual shift forms the foundation of the IL-6–IL-6R homeostasis model proposed in this review. Rather than viewing IL-6 biology solely through the lens of cytokine abundance, this framework considers inflammatory outcomes as the integrated consequence of cytokine production, receptor availability, buffering capacity, and cytokine persistence. We suggest that disruption of any of these regulatory layers may alter the threshold between protective antiviral immunity and pathological inflammation, providing a unifying mechanism to explain inter-individual differences in susceptibility to severe dengue and potentially other inflammatory diseases.

## Dendritic cell-derived IL-6 buffering: a physiological mechanism for cytokine homeostasis

Although cytokine production has traditionally been considered the principal determinant of inflammatory activity, accumulating evidence indicates that cytokines continue to undergo dynamic regulation after secretion. Once released into the circulation, IL-6 enters a highly regulated extracellular environment where soluble receptors, receptor shedding, cellular uptake, vascular distribution, and cytokine clearance collectively determine its biological availability. These post-secretory processes represent an additional layer of immune regulation that complements transcriptional control and may be equally important for preserving immune homeostasis. Consequently, understanding IL-6 biology requires consideration of both cytokine production and the mechanisms that regulate cytokine persistence after secretion.

A major advance in this field came from the demonstration that conventional dendritic cells (cDCs) function as systemic regulators of circulating IL-6 through continuous production of soluble IL-6 receptor (sIL-6R), thereby establishing a physiological blood-buffering system for IL-6. Rather than serving solely as professional antigen-presenting cells, cDCs were shown to maintain systemic IL-6 homeostasis by generating a circulating pool of sIL-6R capable of dynamically interacting with IL-6. Experimental depletion of cDCs or IL-6R conditional knockout in cDCs resulted in a profound reduction in circulating sIL-6R, delayed clearance of IL-6 from the bloodstream, and impaired maintenance of systemic IL-6 concentrations despite preserved cytokine production (39). These findings established that dendritic cells regulate not only immune activation but also the extracellular kinetics of IL-6, identifying a previously unrecognized physiological mechanism governing cytokine homeostasis.

Importantly, this buffering system should not be interpreted simply as an anti-inflammatory pathway. Instead, it functions as a homeostatic rheostat that stabilizes IL-6 availability during acute immune responses. Reversible interactions between IL-6 and soluble IL-6 receptor may transiently preserve circulating cytokine, moderate abrupt fluctuations in free IL-6 concentrations, and coordinate the spatial and temporal distribution of IL-6 signaling. Under physiological conditions, this mechanism may ensure sufficient cytokine availability to support effective antimicrobial immunity while simultaneously preventing excessive or uncontrolled signaling. Conversely, when IL-6 production exceeds the buffering capacity of the soluble receptor pool, free cytokine concentrations may increase sufficiently to amplify inflammatory signaling, promote endothelial activation, and initiate pathological immune responses.

These observations introduce a previously underappreciated dimension of IL-6 biology by distinguishing cytokine production from cytokine persistence. Whereas transcriptional regulation determines the quantity of IL-6 released during the initial inflammatory response, post-secretory buffering mechanisms regulate the duration, distribution, and bioavailability of IL-6 after secretion. Together, these complementary processes determine the overall magnitude of IL-6 signaling and, ultimately, the balance between protective immunity and immunopathology.

The dendritic cell-derived buffering system, therefore, provides the experimentally established physiological foundation for the conceptual framework developed in this review. Building upon these findings, we propose that naturally occurring human genetic variation within the IL6–IL6R axis modifies this conserved homeostatic mechanism. Specifically, regulatory variants within IL-6, such as rs2069843 and rs1800795, are predicted to influence the initial magnitude of cytokine production, whereas functional variation within IL-6R, particularly the rs2228145 (Asp358Ala) polymorphism, may alter receptor shedding, circulating sIL-6R availability, and consequently the efficiency of IL-6 buffering. Importantly, the buffering mechanism itself is independent of rs2228145 and has been experimentally demonstrated without reference to this polymorphism. Rather, we propose that rs2228145 functions as a genetic modifier superimposed upon this conserved physiological system, altering its capacity to regulate IL-6 persistence during acute inflammation.

This distinction forms the central hypothesis of the present review. The dendritic cell-derived IL-6 buffering system represents the established biological mechanism, whereas the integration of IL6R rs2228145 into this framework provides a mechanistic explanation for how inherited variation in receptor biology may influence IL-6 homeostasis during dengue virus infection. Within this IL-6–IL-6R homeostasis model, disease severity is proposed to arise from the interaction between two complementary regulatory axes: the amplitude of cytokine production, largely determined by IL-6 regulatory variation, and the duration of cytokine persistence, governed by soluble receptor biology and modified by IL-6R genetics. We hypothesize that disruption of either regulatory layer or, more importantly, their combined imbalance may shift IL-6 signaling beyond the threshold of physiological homeostasis, promoting sustained endothelial activation, vascular leakage, and progression to severe dengue.

More broadly, this model extends beyond dengue to suggest that cytokine biology should be understood not solely in terms of cytokine synthesis but as the coordinated regulation of cytokine production, receptor availability, buffering capacity, persistence, and signal duration (36, 92). This perspective provides a general framework for investigating how host genetics shapes immune homeostasis across infectious and inflammatory diseases and generates experimentally testable hypotheses for future studies integrating immunogenetics with cytokine physiology.

## Soluble IL-6 receptor biology and the dual nature of classical and trans-signaling

The biological activity of IL-6 is determined not only by cytokine abundance but also by the availability and distribution of its receptors (19, 73, 93–96). Unlike many cytokines, IL-6 signals through a two-component receptor system comprising the ligand-binding interleukin-6 receptor (IL-6R/CD126) and the ubiquitously expressed signal-transducing receptor glycoprotein 130 (gp130/CD130). This receptor architecture enables IL-6 to initiate signaling through two mechanistically distinct but biologically interconnected pathways, classical signaling and trans-signaling, thereby expanding the functional diversity of IL-6 across different tissues and inflammatory contexts.

In classical signaling, IL-6 binds membrane-bound IL-6R (mIL-6R), which is expressed on a restricted subset of cells, including hepatocytes, neutrophils, monocytes, macrophages, and selected lymphocyte populations. Formation of the IL-6/mIL-6R complex promotes gp130 homodimerization and activation of intracellular JAK–STAT3, MAPK, and PI3K–AKT signaling pathways. These cascades regulate acute-phase protein synthesis, B-cell maturation, T follicular helper cell differentiation, plasma cell survival, and tissue regeneration. Consequently, classical signaling is generally regarded as the principal pathway supporting physiological immune responses and restoration of tissue homeostasis following infection (13, 53, 54).

In contrast, trans-signaling arises when membrane IL-6R is proteolytically cleaved by the metalloproteases ADAM10 and ADAM17, generating soluble IL-6 receptor (sIL-6R). Because sIL-6R retains full ligand-binding capacity, circulating IL-6 readily forms IL-6/sIL-6R complexes capable of activating gp130 on virtually all nucleated cells, including vascular endothelial cells that normally lack membrane IL-6R. Through this mechanism, trans-signaling greatly expands the cellular targets of IL-6 and has been implicated in endothelial activation, leukocyte recruitment, vascular permeability, chronic inflammation, and multiple inflammatory diseases (14, 17, 55, 97). These observations have established trans-signaling as a major contributor to pathological inflammation and have provided the rationale for therapeutic strategies selectively targeting the IL-6/sIL-6R axis (53, 98–101).

Although classical and trans-signaling are often presented as opposing pathways, their relationship is better viewed as complementary components of a dynamic receptor network. The generation of sIL-6R simultaneously reduces membrane receptor density while increasing the circulating soluble receptor pool, thereby altering both the cellular distribution and extracellular kinetics of IL-6 (102). Consequently, receptor shedding not only influences which cells can respond to IL-6 but may also regulate the persistence, spatial distribution, and bioavailability of the cytokine itself. This broader perspective extends the role of sIL-6R beyond that of a simple signaling co-receptor and suggests that soluble receptor biology contributes to the maintenance of systemic cytokine homeostasis.

This concept aligns with the experimentally established dendritic cell-derived IL-6 buffering mechanism discussed in the previous section. Within that framework, the soluble IL-6 receptor participates in regulating circulating IL-6 after secretion by influencing cytokine persistence and extracellular availability. Importantly, this physiological buffering mechanism does not negate the well-established pro-inflammatory functions of trans-signaling. Rather, it suggests that the biological consequences of sIL-6R are context-dependent, reflecting a balance between its capacity to preserve circulating IL-6 and its ability to facilitate signaling through gp130-expressing cells. During physiological immune responses, buffering may stabilize IL-6 concentrations and support-controlled cytokine distribution. Conversely, when IL-6 production exceeds buffering capacity or when signaling competent IL-6/sIL-6R complexes accumulate, trans-signaling may become the dominant pathway driving endothelial dysfunction and systemic inflammation.

This duality provides a mechanistic foundation for understanding how inherited variation in IL-6R may influence inflammatory disease. Functional polymorphisms that alter receptor shedding and circulating sIL-6R concentrations are predicted not only to modify the balance between classical and trans-signaling but also to influence the efficiency of the physiological buffering system. Accordingly, the common IL-6R rs2228145 (Asp358Ala) variant may represent a naturally occurring genetic modifier of IL-6 homeostasis, linking receptor biology to inter-individual differences in cytokine persistence, inflammatory signaling, and susceptibility to severe dengue.

## Dengue as a model of dysregulated IL-6 homeostasis

Among acute viral infections, dengue virus (DENV) infection provides a particularly informative model for investigating how dysregulated cytokine homeostasis contributes to immunopathology. DENV infects hundreds of millions of people every year and is still a major public health problem around the world (103, 104). While most infections are asymptomatic or manifest as uncomplicated dengue fever, a subset of patients rapidly progresses to severe dengue, characterized by profound plasma leakage, hemorrhage, shock, and, in some cases, multi-organ failure (41, 47). Severe disease typically develops during the critical phase following the decline of viremia, suggesting that host immune responses, rather than direct viral cytopathic effects, are the principal drivers of tissue injury (41, 44, 46–49).

Accumulating evidence identifies IL-6 as one of the key inflammatory mediators associated with severe dengue (41, 71, 72). Numerous clinical studies have demonstrated that circulating IL-6 concentrations correlate with disease severity, vascular permeability, thrombocytopenia, coagulopathy, and adverse clinical outcomes (40, 43, 45, 72, 84, 105). Mechanistically, IL-6 promotes endothelial activation through JAK-STAT3 signaling, induces expression of vascular permeability mediators such as vascular endothelial growth factor (VEGF), enhances leukocyte recruitment by upregulating adhesion molecules, and amplifies inflammatory cascades through interactions with other cytokines, including TNF-α, IL-1β, and IL-8. Collectively, these processes contribute to disruption of endothelial barrier integrity, a defining pathological feature of severe dengue.

Despite the consistent association between elevated IL-6 levels and disease severity, circulating cytokine concentration alone does not adequately explain the marked heterogeneity of clinical outcomes. Patients with comparable viral loads or similar IL-6 concentrations may exhibit strikingly different clinical trajectories, indicating that additional regulatory mechanisms influence the biological consequences of IL-6 signaling. These observations suggest that the duration of cytokine exposure, receptor availability, downstream signaling capacity, and host genetic variation collectively shape the inflammatory response.

Recent advances in IL-6 biology provide a framework for interpreting this variability. Beyond regulating cytokine production, physiological mechanisms that control IL-6 persistence and bioavailability may critically determine whether inflammatory signaling resolves appropriately or progresses toward sustained endothelial activation and vascular dysfunction. Within this context, dengue serves as a valuable model for examining how inherited variation in both cytokine expression and receptor biology influences immune homeostasis during acute viral infection.

In the following sections, we examine two complementary genetic components of the IL-6 signaling axis. First, we discuss the IL6 rs2069843 and rs1800795 polymorphisms, which is proposed to influence the magnitude of IL-6 production. We then examine the functional IL6R rs2228145 (Asp358Ala) variant, which alters soluble IL-6 receptor generation and may regulate cytokine persistence through receptor-mediated buffering mechanisms. Together, these complementary pathways form the basis of a unified IL-6–IL-6R homeostasis model, in which the balance between cytokine production and cytokine buffering determines susceptibility to protective immunity or immunopathology during dengue virus infection.

## IL6 rs2069843 and rs1800795: genomic organization and molecular biology

The *IL6* gene is located on chromosome 7p15.3 and spans approximately 6 kb, comprising five exons and four introns that encode the multifunctional cytokine interleukin-6 (IL-6) (106, 107). IL-6 is produced by a broad spectrum of immune and non-immune cells, including monocytes, macrophages, dendritic cells, neutrophils, endothelial cells, fibroblasts, epithelial cells, and activated lymphocytes (108, 109). Its expression is tightly regulated by inflammatory stimuli and environmental cues, reflecting the requirement for rapid yet transient cytokine production during host defense. The *IL6* locus contains numerous regulatory elements within both promoter and intronic regions, allowing integration of multiple signaling pathways that coordinate transcriptional activation in response to infection or tissue injury (82, 90, 110).

Among the genetic variants identified within IL6, the intronic single nucleotide polymorphism rs2069843 has emerged as a candidate susceptibility locus for inflammatory diseases, including dengue virus infection (85, 111–114). Although this variant does not alter the amino acid sequence of IL-6, increasing evidence indicates that intronic polymorphisms can exert important regulatory functions through mechanisms affecting chromatin accessibility, transcription factor binding, enhancer activity, RNA polymerase II recruitment, alternative splicing, and interactions with non-coding RNAs (115–120). Consequently, non-coding variants are increasingly recognized as important determinants of inter-individual differences in cytokine expression rather than biologically neutral polymorphisms (121–124).

The precise molecular function of rs2069843 has not yet been experimentally established. However, its genomic location within a regulatory intronic region raises the possibility that it influences *IL6* transcription by modifying local cis-regulatory architecture. One plausible mechanism is that the variant alters enhancer activity or transcription factor occupancy, thereby influencing the magnitude of *IL6* transcription following activation of inflammatory pathways such as NF-κB, AP-1, and C/EBPβ (119). Alternatively, rs2069843 may exist in linkage disequilibrium with other functional regulatory variants that collectively shape *IL6* expression (125). Future studies integrating expression quantitative trait locus (eQTL) analyses, chromatin accessibility profiling, transcription factor occupancy mapping, and CRISPR-based genome editing will be required to define the causal mechanism underlying this association.

Unlike rs2069843, which is located within an intronic regulatory region, the *IL6* rs1800795 polymorphism (also known as −174 G>C) resides in the promoter of the *IL6* gene, approximately 174 base pairs upstream of the transcription start site on chromosome 7p15.3. The *IL6* rs1800795 is one of the most extensively investigated functional variants within the *IL6* gene (126, 127). Owing to its location within a key cis-regulatory element, rs1800795 has the potential to alter the binding affinity of transcription factors that regulate *IL6* transcription during inflammatory responses (120). The promoter region surrounding rs1800795 contains multiple regulatory motifs responsive to inflammatory and hormonal signals, including binding sites for NF-κB, CCAAT/enhancer-binding proteins (C/EBPs), NF-IL-6 (C/EBPβ), AP-1, and the glucocorticoid receptor (GR) (128–131). Although rs1800795 does not directly disrupt all of these binding motifs, the G-to-C substitution is thought to modify the local chromatin architecture and DNA–protein interactions, thereby influencing transcription factor recruitment and promoter accessibility. Experimental studies have shown that the two alleles exhibit differential promoter activity in reporter assays, with the G allele generally associated with higher transcriptional activity and greater IL-6 production following inflammatory stimulation, whereas the C allele is more frequently associated with reduced promoter activity and lower cytokine expression (79, 82, 132). Consequently, rs1800795 contributes to inter-individual variability in both basal and inducible IL-6 expression and has emerged as an important genetic determinant of susceptibility, disease severity, and clinical outcomes across a broad spectrum of inflammatory, autoimmune, cardiovascular, and infectious diseases, including dengue virus infection (87, 88, 133–135).

From a functional perspective, regulatory variation within IL-6 is expected to influence the quantity of cytokines produced during the initial inflammatory response, thereby modifying the amplitude of downstream immune activation. Even modest alterations in IL-6 expression may have substantial biological consequences because IL-6 functions near the apex of inflammatory signaling networks, where small differences in cytokine production can be amplified through positive feedback loops involving TNF-α, IL-1β, chemokines, endothelial activation, and acute-phase responses. Such amplification is particularly relevant during acute viral infections, where the balance between effective pathogen control and excessive inflammation is exceptionally narrow (16, 23, 136, 137).

Within the conceptual framework proposed in this review, IL-6 rs2069843 and rs1800795 represent the first regulatory layer governing IL-6 homeostasis by determining the magnitude of cytokine production immediately following innate immune activation. However, differences in IL-6 production alone are unlikely to account for the marked heterogeneity in clinical outcomes observed in inflammatory diseases such as dengue (45, 84, 138, 139). The biological activity of IL-6 also depends on the duration of cytokine availability, receptor dynamics, and tissue responsiveness. These additional regulatory dimensions are largely determined downstream of cytokine synthesis and form the basis for the complementary role of IL-6R genetics discussed in subsequent sections. We therefore propose that *IL6* rs2069843 and rs1800795 should be viewed not as an isolated susceptibility variant but as one component of a broader, multi-layered regulatory network in which cytokine production, receptor biology, and cytokine persistence collectively determine IL-6-mediated immune homeostasis.

## *IL6R* rs2228145 (Asp358Ala): a functional genetic modifier of IL-6 homeostasis

Among the common genetic variants affecting the IL-6 signaling axis, *IL6R* rs2228145 (Asp358Ala; formerly rs8192284) is one of the most extensively characterized functional polymorphisms. This nonsynonymous single nucleotide variant results in the substitution of aspartic acid with alanine at position 358 within the extracellular domain of IL-6R gene on chromosome 1q21.3, immediately adjacent to the principal proteolytic cleavage site recognized by the metalloproteases ADAM10 and ADAM17. Multiple genetic, biochemical, and epidemiological studies have consistently demonstrated that the 358Ala allele enhances ectodomain shedding of membrane-bound IL-6R, leading to reduced surface receptor expression and increased circulating concentrations of soluble IL-6 receptor (sIL-6R) (91, 97, 102, 140, 141). These molecular effects have been replicated across diverse populations, establishing rs2228145 as a bona fide functional variant that alters receptor biology rather than serving merely as a genetic marker.

The immunological consequences of enhanced receptor shedding are multifaceted. Reduced membrane IL-6R expression is predicted to diminish classical IL-6 signaling in receptor-expressing cells, whereas elevated circulating sIL-6R expands the extracellular receptor pool capable of binding IL-6 (140). Historically, the biological significance of rs2228145 has been interpreted primarily through its effects on IL-6 trans-signaling, whereby IL-6/sIL-6R complexes activate gp130-expressing cells lacking membrane IL-6R, particularly vascular endothelial cells. This paradigm has substantially advanced understanding of IL-6 biology and underpins the development of therapeutic strategies targeting the IL-6–IL-6R axis. However, observations that rs2228145 exhibits protective effects in some inflammatory disorders while conferring risk or neutral effects in others indicate that the biological consequences of increased sIL-6R cannot be explained solely by enhanced trans-signaling (142–147).

An important advance in understanding this apparent paradox came from the identification of a dendritic cell-derived soluble receptor buffering system that regulates systemic IL-6 homeostasis. This experimentally established mechanism demonstrated that conventional dendritic cells continuously generate circulating sIL-6R, thereby influencing the persistence and systemic availability of IL-6 after secretion (39). Importantly, this physiological buffering mechanism was defined independently of human genetic variation and therefore represents a conserved regulatory process rather than a consequence of the rs2228145 polymorphism.

In dengue, where excessive IL-6 production is consistently associated with endothelial dysfunction, vascular leakage, and severe clinical manifestations, alterations in IL-6 receptor biology may substantially influence the magnitude and duration of inflammatory responses. Although genetic association studies of IL6R rs2228145 (Asp358Ala) in dengue remain limited, available evidence is consistent with a protective role for the 358Ala (C) allele. Individuals carrying the C allele have been reported to exhibit a lower risk of severe dengue, whereas the ancestral A allele has been associated with more pronounced clinical manifestations (141, 148). This observation is biologically plausible because the C allele increases shedding of the membrane-bound IL-6 receptor, resulting in higher circulating concentrations of soluble IL-6 receptor (sIL-6R) and reduced membrane IL-6R expression (149). These changes may attenuate IL-6 classical signaling while modifying cytokine bioavailability, thereby influencing the balance between cytokine buffering and tissue responsiveness.

Building upon this physiological foundation, we propose that *IL6R* rs2228145 functions as a genetic modifier of an evolutionarily conserved IL-6 homeostatic system. Specifically, the increased receptor shedding associated with the 358Ala allele is predicted to expand the circulating soluble receptor pool, thereby modifying the buffering capacity of the extracellular compartment. Within this framework, rs2228145 does not create the buffering mechanism but instead influences its efficiency by altering soluble receptor availability. Consequently, the functional impact of the variant extends beyond determining which cells can respond to IL-6 and may also influence how long IL-6 remains biologically available, how it is distributed within the circulation, and how effectively cytokine concentrations are stabilized during acute inflammation.

This model provides a conceptual reconciliation of the dual biological roles of the soluble IL-6 receptor. Under physiological conditions or during moderate inflammatory responses, increased sIL-6R may enhance homeostatic buffering, stabilize circulating IL-6 concentrations, and contribute to controlled immune regulation. In contrast, when IL-6 production exceeds the buffering capacity of the soluble receptor pool, accumulation of signaling-competent IL-6–sIL-6R complexes may preferentially promote gp130-mediated trans-signaling, resulting in endothelial activation, vascular dysfunction, and amplification of inflammatory cascades. Thus, the biological consequences of rs2228145 are proposed to depend not simply on receptor abundance but on the dynamic relationship between cytokine production, buffering capacity, sgp130 and the threshold at which homeostatic regulation transitions to pathological signaling.

Within the IL-6–IL-6R homeostasis model proposed in this review, rs2228145 therefore represents the second major regulatory axis governing IL-6 biology. Whereas *IL6* rs2069843 and rs1800795 are proposed to influence the amplitude of cytokine production, *IL6R* rs2228145 may regulate the persistence, bioavailability, and extracellular kinetics of IL-6 through its effects on soluble receptor dynamics. Together, these complementary mechanisms determine the overall magnitude and duration of IL-6 signaling, thereby shaping susceptibility to either protective antiviral immunity or pathological inflammation (**Figure 2**).

**Figure 2:**
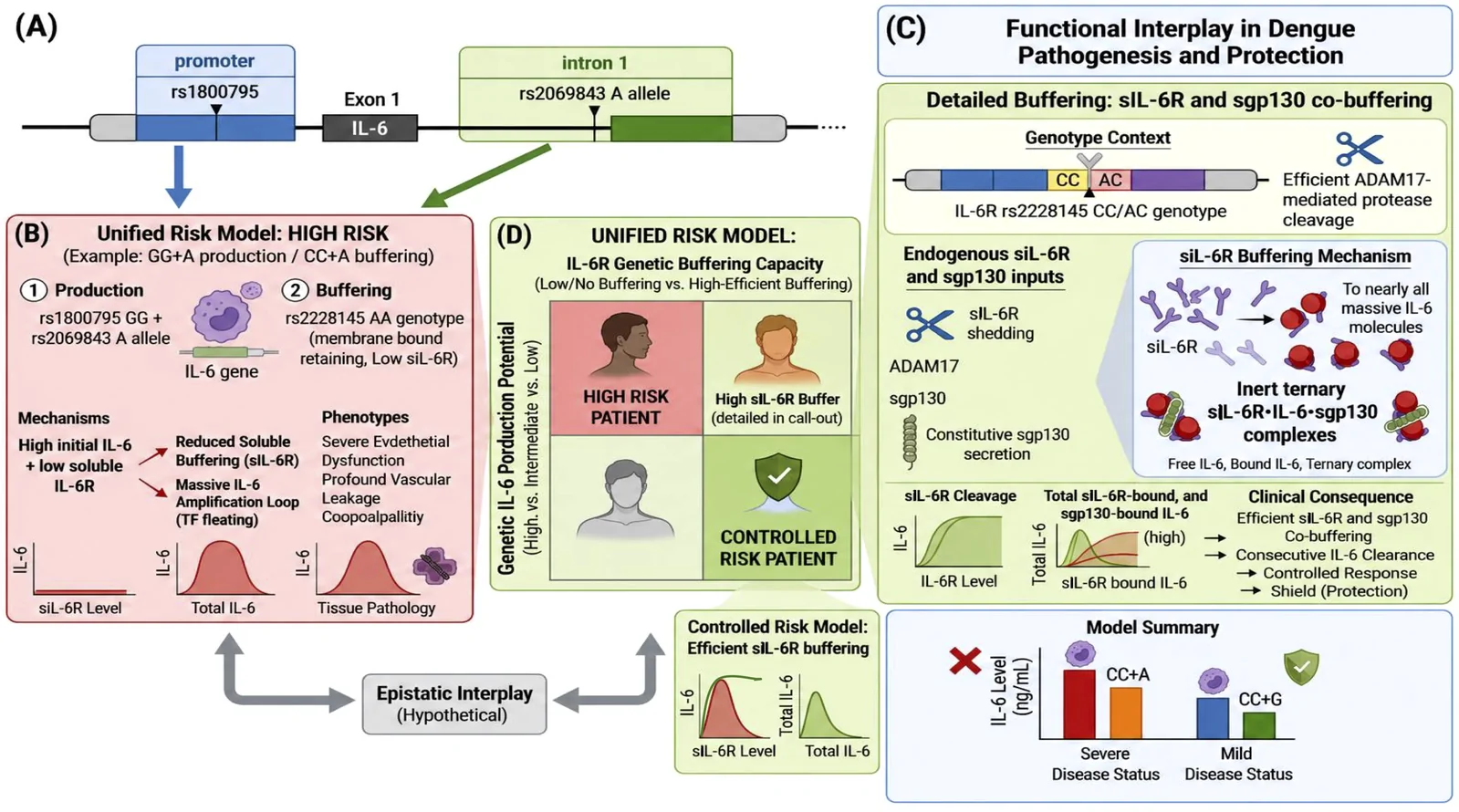
Unified model of genotype-dependent interplay between IL6 production and IL6R buffering in dengue pathogenesis. This composite figure integrates genetic architecture, cellular mechanisms, and risk stratification to demonstrate how polymorphisms in *IL6* and *IL6R* determine the disease severity. **(A)** *IL6* gene structure with promoter (blue) and intron (green) polymorphic sites (arrows indicating ‘zoom’ influence) directing basal production potential. **(B)** High-risk scenario workflow initiated by high-production *IL6* genotypes paired with a low-buffering *IL6R* genotype, detailing downstream pathogenic mechanisms (arrows) and clinical phenotypes, supported by kinetic curves of IL-6 levels and tissue pathology (graphs). **(C)** Mechanisms of functional protection through robust buffering, contrasting low-sIL6R/sgp130 inputs with high inputs, and demonstrating ternary sIL6R–IL6–sgp130 complex formation, supported by clearance kinetics and shielding outcomes. **(D)** A 2 × 2 matrix-based clinical risk model integrating genetic production potential with genetic buffering capacity to categorize patients as high-risk or controlled risk, supported by a model summary of severe vs. mild disease outcomes based on specific genotype pairs.

It is important to emphasize that this integrated model remains hypothesis-driven. Although compelling evidence demonstrates that rs2228145 enhances IL-6R shedding and increases circulating sIL-6R, direct evidence that the variant modifies IL-6 persistence during dengue virus infection is currently unavailable. Accordingly, we propose this framework as a testable mechanistic hypothesis rather than a definitive explanation of dengue immunopathogenesis. Future studies integrating genotype-stratified dengue cohorts with longitudinal measurements of IL-6, sIL-6R, soluble gp130, receptor occupancy, cytokine half-life, and endothelial biomarkers, together with experimental models employing allele-specific genome editing, will be essential to determine whether rs2228145 reshapes systemic IL-6 homeostasis during acute viral infection.

Viewed from this perspective, *IL6R* rs2228145 represents more than a functional receptor polymorphism. It constitutes a naturally occurring experiment in human immunology that links receptor shedding, soluble receptor biology, and cytokine homeostasis. By integrating this variant with the experimentally established dendritic cell-derived buffering mechanism, we propose a unified framework in which inherited variation in receptor biology modifies a conserved physiological system, thereby influencing the balance between protective antiviral immunity and immunopathology. Although developed in the context of dengue virus infection, this conceptual model may have broader implications for understanding IL-6 regulation across viral infections, autoimmune diseases, and other inflammatory disorders.

## Dendritic cell-derived IL-6R buffering: experimental evidence and relevance to dengue

The proposed IL-6 production–IL-6R buffering framework is grounded in experimental evidence demonstrating a physiologically important role for dendritic cell-derived soluble IL-6 receptor (sIL-6R) in regulating systemic IL-6 homeostasis during viral infection. Previous experimental study demonstrated that conventional dendritic cells (cDCs) serve as a major source of circulating sIL-6R, which binds IL-6 and contributes to controlling its systemic availability (39). Importantly, the functional relevance of this buffering mechanism has been demonstrated experimentally in animal models of acute influenza virus infection. Mice infected with either the NC99 or PR8 influenza strains exhibited increased morbidity and mortality when the sIL-6R buffering system was deficient, demonstrating that this pathway plays a protective role during acute viral infection. Moreover, restoration of circulating sIL-6R through adeno-associated virus (AAV)-mediated delivery rescued the deficient phenotype in mice lacking sIL-6R, providing direct functional evidence that sIL-6R-mediated buffering contributes to protection against excessive IL-6-associated immunopathology (39).

These findings indicate that sIL-6R-mediated regulation is not confined to noninfectious or steady-state inflammatory conditions but can become functionally important during acute viral infection. They also provide experimental support for the broader concept that the relationship between cytokine production and soluble receptor-mediated regulation may influence inflammatory disease outcomes. Consistent with this possibility, genetic association studies in other viral diseases have reported relationships between IL6 and IL6R variants and clinical outcomes. In COVID-19, several studies have reported associations between the IL6 rs1800795 G allele or GG genotype and disease severity or mortality (150–154). Similarly, IL6R rs2228145 has been associated with post-acute COVID-19 outcomes, with the AA genotype reportedly associated with a higher risk of long COVID than the C allele (93). In chronic hepatitis C and B virus infection, studies have also reported associations between IL6 rs1800795 and disease susceptibility, persistence, or severity, although the direction and magnitude of these associations have varied across populations and clinical settings (134, 155–160). In HCV infection, the IL6R rs2228145 AA genotype has additionally been associated with increased viremia and viral persistence (161).

Importantly, these genetic associations should not be interpreted as direct evidence that IL-6R buffering mediates the reported disease phenotypes. IL6 and IL6R polymorphisms can influence cytokine production, receptor expression or shedding, and downstream signaling in context-dependent ways, and associations reported in different viral infections have not been uniformly replicated. Nevertheless, together with the experimental influenza data, these observations support the rationale for investigating whether genetically determined differences in IL-6 production and soluble IL-6R regulation contribute to inter-individual variation in inflammatory responses to viral infection.

A critical distinction must therefore be maintained between experimentally established mechanisms and their proposed application to dengue. Although influenza models demonstrate that the sIL-6R buffering system can regulate disease outcome during acute viral infection, direct evidence that this system controls IL-6 persistence, inflammatory signaling, endothelial dysfunction, or vascular leakage during acute DENV infection is currently unavailable. In particular, it remains unknown whether differences in circulating sIL-6R, soluble gp130 (sgp130), or their relationship to IL-6 concentrations quantitatively influence the transition from controlled inflammation to the vascular and inflammatory disturbances associated with severe dengue.

Accordingly, our hypothesis does not assume that the influenza mechanism operates identically during dengue. Rather, the influenza findings provide a mechanistic foundation for testing whether a comparable imbalance between IL-6 production and extracellular IL-6R-mediated regulation contributes to the highly dynamic inflammatory environment of acute DENV infection. This question is particularly relevant because severe dengue is characterized by substantial cytokine dysregulation and endothelial dysfunction; however, these clinical manifestations arise from coordinated interactions among multiple inflammatory, vascular, endothelial, and hemostatic pathways and cannot be attributed to IL-6 signaling alone.

Thus, the central distinction of the proposed framework is not the identification of a new IL-6 signaling pathway. Instead, it is the hypothesis that the quantitative balance between IL-6 production and extracellular regulation by sIL-6R and sgp130 may represent an additional layer of systemic control that influences the magnitude and persistence of IL-6 activity during acute viral infection. The existence and protective function of sIL-6R buffering during acute influenza infection have been experimentally demonstrated, whereas its quantitative contribution and disease-specific consequences during dengue and other viral infections remain to be established. This distinction defines a testable research framework rather than an established mechanism of dengue pathogenesis.

## Role of soluble gp130 in trans-signaling and IL-6R buffering

Soluble gp130 (sgp130) represents an additional and potentially critical regulatory layer within the IL-6-IL-6R model because it selectively neutralizes IL-6/sIL-6R complexes and thereby limits IL-6 trans-signaling (162, 163). In this context, circulating sIL-6R and sgp130 should not be considered independent biomarkers but rather components of an extracellular buffering network that determines the fraction of IL-6 that remains available to engage membrane gp130 on IL-6R-negative cells. Under homeostatic conditions, sIL-6R can extend the biological range of IL-6, whereas abundant circulating sgp130 can sequester IL-6/sIL-6R complexes and constrain trans-signaling. Thus, the functional outcome of increased sIL-6R availability is likely to depend not only on the amount of IL-6 produced but also on the relative abundance and binding capacity of sgp130 (56).

This relationship provides a mechanistic basis for considering sgp130 as a potential boundary condition between physiological IL-6 buffering and excessive trans-signaling. When IL-6/sIL-6R complex formation remains within the neutralizing capacity of circulating sgp130, trans-signaling may be effectively buffered despite increased sIL-6R availability. Conversely, when IL-6 production and/or sIL-6R availability increases sufficiently to exceed the effective neutralization capacity of sgp130, an increasing proportion of IL-6/sIL-6R complexes may remain available to interact with membrane gp130, potentially expanding trans-signaling to cells that do not express membrane IL-6R. The relevant determinant is therefore not an absolute concentration of sIL-6R alone, but the quantitative relationship among IL-6, sIL-6R, and sgp130, together with their binding affinities, molecular stoichiometry, and rates of production and clearance.

Importantly, the term “thermodynamic boundary” should be interpreted as a conceptual rather than a fixed biological threshold. The transition from predominantly buffered to increasingly active trans-signaling is unlikely to occur at a single universal concentration of IL-6 or sIL-6R. Instead, it should depend on the relative concentrations and binding equilibria of IL-6, sIL-6R, and sgp130, as well as the kinetics of complex formation, receptor engagement, and clearance.

In acute viral infection and severe dengue, this framework may be particularly relevant because simultaneous increases in inflammatory cytokine production and alterations in receptor shedding could challenge the available sgp130 buffering capacity. However, whether circulating sgp130 levels vary sufficiently during dengue infection to shift the balance from predominantly buffered IL-6/sIL-6R complexes toward pathological trans-signaling remains an important unanswered question. Longitudinal measurement of IL-6, sIL-6R, and sgp130, ideally together with direct or inferred measurements of IL-6/sIL-6R complex formation and downstream trans-signaling activity, would therefore provide a more informative assessment of IL-6 system dynamics than measurement of any individual component alone. Such studies could determine whether inter-individual variation in sgp130 represents an additional determinant of susceptibility to sustained IL-6 trans-signaling and endothelial or inflammatory pathology.

## Integration of IL-6–IL-6R buffering with broader cytokine regulatory networks

Immune homeostasis during viral infection is governed by highly interconnected cytokine and intracellular signaling networks rather than by isolated pathways. Accordingly, the proposed IL-6 production–IL-6R buffering framework should not be interpreted as an independent mechanism controlling inflammation, but rather as one regulatory layer within a broader network that determines the magnitude, duration, and cellular consequences of cytokine signaling. IL-6 production and activity are dynamically influenced by interactions with IL-10, NF-κB, interferon pathways, SOCS3/STAT3 feedback, and T-cell differentiation programs, all of which may modify the biological consequences of altered IL-6 availability (12, 18, 19, 130).

At the level of inflammatory cytokine production, NF-κB represents an important upstream regulatory pathway linking innate immune sensing to the transcriptional induction of IL-6 and other inflammatory mediators. Viral recognition and tissue inflammation can activate NF-κB through multiple pattern recognition receptor pathways, thereby promoting IL-6 production along with TNF-α, IL-1β, and other inflammatory mediators. In this context, genetic or environmental factors that increase IL-6 production should be considered within a coordinated inflammatory response, rather than as isolated determinants of cytokine abundance. Conversely, excessive or sustained NF-κB activation may increase the demand placed on the IL-6R buffering system by elevating systemic IL-6 production.

Once IL-6 engages membrane-bound or soluble IL-6R, signaling is predominantly mediated through gp130 and the activation of the JAK–STAT3 pathway. STAT3 also provides an important negative-feedback mechanism through the induction of suppressor of cytokine signaling 3 (SOCS3), which inhibits proximal signaling downstream of cytokine receptors. Thus, IL-6 activity is regulated not only by the amount of cytokine produced and the availability of IL-6R but also by intracellular feedback mechanisms that determine cellular responsiveness. This provides an important conceptual extension of the production–buffering model: systemic IL-6 concentration and sIL-6R availability may influence the extracellular signaling environment, whereas SOCS3/STAT3 feedback determines, in part, how individual cells translate that extracellular signal into a biological response. Alterations at either level could therefore modify the duration or magnitude of IL-6 signaling without necessarily changing circulating IL-6 concentrations.

IL-10 provides an additional counter-regulatory axis that can restrain inflammatory cytokine production and limit tissue injury. IL-10 suppresses inflammatory responses in multiple myeloid-cell populations and can reduce production of cytokines including IL-6 and TNF-α. The balance between IL-6-driven inflammation and IL-10-mediated negative regulation may therefore be particularly important during acute viral infection. A state characterized by increased IL-6 production together with insufficient counter-regulation could favor persistent inflammation, whereas effective IL-10 responses may limit the consequences of increased IL-6 availability. This reinforces the concept that IL-6 buffering should be viewed as one component of a larger network of mechanisms that collectively maintain inflammatory homeostasis.

Interferon signaling further intersects with the IL-6 pathway during viral infection. Type I and type II interferons activate distinct but interconnected JAK-STAT pathways that promote antiviral gene expression and shape innate and adaptive immune responses. Depending on the cellular and temporal context, interferon signaling can influence IL-6 production, cellular responsiveness, and the composition of the inflammatory environment. Conversely, excessive inflammatory signaling can alter the balance between antiviral interferon responses and inflammatory cytokine pathways. Therefore, differences in IL-6 production or receptor buffering cannot be interpreted independently of the interferon response, particularly during acute DENV infection in which antiviral and inflammatory pathways are simultaneously engaged.

IL-6 signaling also influences adaptive immune differentiation and therefore may contribute to the transition from innate inflammation to longer-term immune responses. In the presence of appropriate cytokine and transforming growth factor-β signals, IL-6 promotes differentiation toward Th17 cells, whereas in other cytokine environments it can influence T follicular helper and other CD4+ T-cell programs. Through these effects, alterations in IL-6 availability may influence not only acute inflammatory responses but also the quality and magnitude of adaptive immunity. However, these effects are strongly dependent on cytokine context and cellular environment and should not be interpreted as direct consequences of IL-6 concentration alone.

Taken together, these interactions suggest that the biological effect of an IL-6 genetic variant can not be predicted solely from its effect on cytokine production, nor can the consequence of *IL6R* rs2228145 be inferred solely from its effect on receptor shedding. The functional phenotype is likely determined by the interaction among IL-6 production, sIL-6R-mediated extracellular buffering, gp130 signaling, SOCS3/STAT3 negative feedback, IL-10-mediated suppression, NF-κB-driven inflammatory activation, interferon signaling, and downstream effects on immune-cell differentiation. The proposed IL-6 production-IL-6R buffering model therefore represents one level of regulation within a multi-layered cytokine network rather than a standalone explanation of immune homeostasis or dengue pathogenesis.

In the context of dengue, this integrated perspective is particularly important because disease severity reflects the coordinated interactions among inflammatory cytokines, antiviral pathways, endothelial responses, complement activation, platelet dysfunction, and adaptive immunity. The central hypothesis of this review is consequently not that IL-6-IL-6R signaling independently determines disease outcome, but that variation in IL-6 production and soluble receptor buffering may alter one important regulatory node within this interconnected network.

## *IL6* and *IL6R* polymorphisms in dengue: evidence and population variability

The reported associations between IL6/IL6R polymorphisms and dengue susceptibility or disease severity should be interpreted cautiously, as the available evidence remains incompletely replicated across populations. Although *IL6* rs2069843, *IL6* rs1800795, and *IL6R* rs2228145 have been proposed as potentially important determinants of the host IL-6 response, current evidence does not support considering any of these variants as universal or independent genetic determinants of severe dengue (**Table 1**).

**Table 1:** Candidate genetic modifiers of IL-6/IL-6R biology and dengue clinical outcomes.

Variant	Gene	Variant allele	Reported association	Population	Sample size	Dengue outcome	Conflicting evidence	Key limitations	Ref
rs2069843	*IL6*	A	Associated with severity/susceptibility	Colombia	266	↑	No	Ethnicity, stratification, Sample size, replication	(85)
rs1800795	*IL6*	C	Reduced susceptibility/protection to hemorrhagic fever	Brazil	200	↓	No	Ethnicity, stratification, Sample size, replication	(164)
		C	Reduced susceptibility/protection	Brazil	337	↓	No	Ethnicity, stratification	(132)
		C	Association with persistence of dermatological symptoms	Brazil	96	↑	Yes	Ethnicity, stratification, Sample size, replication	(165)
		C/G	Not associated with hemorrhagic fever	Brazil	198	None	Yes	Ethnicity, stratification, Sample size, replication	(166)
		C	Not associated with hemorrhagic fever	Cuba	43	None	No	Ethnicity, stratification, Sample size, replication	(167)
		C	Not associated with hemorrhagic fever	Venezuela	66	None	No	Ethnicity, stratification, Sample size, replication	(168)
rs2228145	*IL6R*	AA	Associated with clinical dengue	European, Amerindian and African	298	↑	No	Ethnicity, stratification, Sample size, replication	(141)
		C	Reduced susceptability/prtoection against dengue	Afro-Colombian and Amerindian	191	↓	No	Ethnicity, stratification, Sample size, replication	(141)

↑, increased susceptibility and disease severity; ↓ reduced susceptibility and disease severity.

For *IL6* rs2069843, evidence from a Colombian cohort suggested an association between the A allele and dengue hemorrhagic fever, including associations that remained evident after adjustment for genetic ancestry (85). These findings provide a plausible indication that rs2069843 may influence the magnitude of the IL-6 response during dengue infection. However, the evidence is derived primarily from relatively limited, population-specific studies, and independent replication in other ethnic and geographic populations is required before this association can be considered generalizable. Similarly, studies of the IL6 promoter variant rs1800795 have reported associations with dengue severity. A meta-analysis of available studies suggested that the CC/GC genotypes were associated with a reduced risk of more severe dengue phenotypes, whereas the GG genotype was more frequently observed among patients with symptomatic dengue (88, 164). Consistent with its proposed functional effect, individuals carrying the CC/GC genotypes have also been reported to exhibit lower IL-6 production than GG carriers (155). However, the meta-analysis included only a small number of studies, involving patients primarily from Brazil, Cuba, and Venezuela, thereby limiting statistical power and the ability to establish the consistency of these associations across populations. Thus, the direction and magnitude of associations involving IL6 variants may be context-dependent rather than uniform across populations.

The established functional effect of *IL6R* rs2228145 on IL-6R shedding and the resulting increase in circulating soluble IL-6R (sIL-6R) provides a strong biological rationale for considering this variant as a potential modifier of IL-6 signaling and extracellular receptor buffering. Importantly, a study in a Colombian cohort reported that individuals carrying the CC/AC genotypes had lower susceptibility to dengue and that these genotypes were associated with the highest circulating sIL-6R concentrations compared to AA genotype. These findings are consistent with the possibility that increased sIL-6R availability may contribute to protection during dengue infection (141, 148). However, the observed genetic association and the established functional effect of rs2228145 should not be interpreted as evidence that increased sIL-6R directly mediates protection from dengue. Direct evidence linking rs2228145 to dengue severity or demonstrating that this variant alters IL-6 persistence or systemic IL-6 signaling during acute DENV infection, remains limited. Accordingly, its proposed contribution to protection from or susceptibility to severe dengue should currently be regarded as a mechanistically plausible but clinically incompletely validated hypothesis.

Several additional factors complicate the interpretation of cytokine polymorphism studies. Many have relatively small sample sizes, increasing the risk of unstable effect estimates and both false-positive and false-negative findings. Ethnic heterogeneity and population stratification may further confound genetic associations when ancestry is not adequately controlled. In addition, differences in dengue serotype, primary versus secondary infection, age distribution, disease classification, and the timing of cytokine measurement can substantially influence both genotype–phenotype relationships and circulating cytokine concentrations. These considerations are particularly relevant to IL-6, whose production is highly dynamic and regulated by multiple cellular and inflammatory pathways. Consequently, an association observed in one population or clinical setting may not necessarily be reproducible in another.

Taken together, the current literature supports the possibility that *IL6* and *IL6R* genetic variation contributes to inter-individual differences in inflammatory responses to DENV, but the evidence remains insufficient to establish consistent, population-independent effects on dengue severity. Importantly, there is also limited evidence from studies simultaneously evaluating *IL6* and *IL6R* variants within the same dengue cohorts, leaving unresolved whether variation in IL-6 production and variation in IL-6R-mediated buffering interact functionally *in vivo*. We therefore propose the IL-6 production–IL-6R buffering framework as a testable mechanistic model rather than a definitive genetic explanation of dengue pathogenesis.

Future adequately powered, multi-ethnic studies should simultaneously genotype relevant *IL6* and *IL6R* variants, rigorously account for population structure and infection-related covariates, and integrate genotype with longitudinal measurements of IL-6, sIL-6R, soluble gp130, and endothelial and inflammatory biomarkers. Where feasible, such studies should additionally incorporate measurements of receptor occupancy, IL-6 clearance kinetics, and downstream signaling activity. These approaches will be essential to determine the reproducibility, population specificity, and biological significance of these genetic associations and to establish whether particular IL-6-IL-6R genotype combinations influence systemic IL-6 homeostasis and clinical outcomes during dengue and other viral infections.

## Perspective: translating the unified IL-6–IL-6R framework into clinical reality

The conventional view of acute viral hyperinflammation has long suffered from a production-centric bias. In conditions like severe dengue, highly elevated circulating interleukin-6 (IL-6) is routinely documented, yet individual clinical trajectories vary dramatically. By introducing a unified framework that balances transcriptional cytokine production (*IL6* rs2069843, rs1800795) against soluble receptor buffering and persistence (*IL6R* rs2228145) and sgp130, a more nuanced paradigm emerges. This perspective shifts the focus from structural cytokine volume to functional cytokine bioavailability. Translating this model into clinical practice requires a fundamental restructuring of diagnostic biomarkers, therapeutic interventions, and clinical trial designs for acute viral infections.

## Clinical implications

The primary clinical implication of the unified IL-6–IL-6R framework is the transition from a standard “one-size-fits-all” model of inflammatory assessment to a genetically informed, kinetic risk profile. In acute infections like dengue, a single snapshot measurement of total plasma IL-6 provides very little prognostic value. Under the unified model, a patient with high genetic production potential (*IL6* rs2069843, rs1800795 GG high-expression genotype) paired with low buffering capacity (*IL6R* rs2228145 membrane-bound retaining genotype) faces a high risk of sustained cytokine amplification. This specific pairing causes unchecked signaling, driving endothelial barrier breakdown, profound vascular leakage, and coagulopathy. Conversely, a patient exhibiting identical initial total IL-6 levels but possessing an efficient soluble receptor buffering system may experience rapid, controlled cytokine clearance without severe tissue pathology. Clinicians can utilize these dual genetic axes to stratify patients early in the disease course, identifying those prone to rapid decompensation long before clinical warning signs manifest.

## Biomarkers

Transitioning this conceptual framework into the clinic necessitates a new generation of multi-analyte biomarker panels. Measuring total IL-6 alone is no longer sufficient; instead, laboratories must concurrently quantify total IL-6, membrane IL-6-receptor (mIL-6R), soluble IL-6 receptor (sIL-6R), soluble gp130 (sgp130) and cytokine-receptor complex kinetics.

From these measurements, clinicians can calculate a free IL-6 index or a bioavailable IL-6 ratio. This value accurately reflects the unbuffered, pathogenic pool of cytokines actively driving classic trans*-*signaling across the vascular endothelium. Furthermore, integrating low-cost, rapid genetic screening for the *IL6* rs2069843, rs1800795 and *IL6R* rs2228145 variants into standard triage processes in the emergency department would provide an invaluable baseline risk score. Combining these genetic risk alleles with real-time, kinetic measurements of circulating soluble receptors creates a dynamic, high-resolution diagnostic readout of a patient’s immediate immunological trajectory.

## Therapeutics

The unified framework directly paves the way for precision immunomodulatory therapies. Historically, broad anti-IL-6 strategies, such as the monoclonal antibody tocilizumab, have yielded mixed results in acute viral infections. Total blockade can inadvertently suppress vital, protective signaling pathways required for host defense and tissue repair.

By applying the buffering framework, therapies can be precisely tailored to a patient’s specific deficit:

a) For High Production/High Persistence Patients: Instead of completely shutting down cytokine production, therapeutics could mimic the natural buffering system. Administering recombinant sIL-6R or engineered sgp130-Fc fusion proteins (such as olamkicept) could selectively neutralize pathogenic trans-signaling while preserving protective signaling pathways.

b) Genetically Targeted Timing: Patients identified via genetic screening as rapid accumulators would receive ultra-early, short-course IL-6 path inhibitors, preventing the initial endothelial damage wave entirely. This approach shifts the therapeutic goal from crude immunosuppression to precise homeostatic restoration.

## Current controversies

Despite the elegance of the unified model, several critical controversies remain unresolved within the immunology community:

- The Dual Nature of sIL-6R: A primary debate centers on the exact physiological behavior of the soluble IL-6 receptor pool. While the buffering model demonstrates that sIL-6R acts as a reservoir to safely control cytokine persistence, classical immunology dictates that sIL-6R complexes with IL-6 to drive systemic trans*-*signaling on cells lacking the membrane-bound receptor. Defining the exact quantitative threshold where sIL-6R transitions from a protective buffer to a pathogenic amplifier represents a critical knowledge gap.
- Tissue-Specific vs. Systemic Dynamics: Most human genetic data relies entirely on peripheral blood measurements. It remains highly controversial whether circulating serum dynamics accurately reflect the localized microenvironment of the lymphoid organs or the microvascular endothelial beds where severe dengue pathology actually takes place.

## Limitations and future directions

An important limitation of the proposed framework is the absence of direct experimental evidence linking the two genetic axes IL-6 production and IL-6R-mediated buffering within the same biological system. The model proposes that variation in the *IL6* locus may influence the magnitude of IL-6 production, whereas *IL6R* rs2228145 may alter receptor shedding and thereby modify the extracellular buffering and signaling capacity of the IL-6 system. However, these relationships have largely been investigated independently, and evidence from studies in which *IL6* and *IL6R* genotypes were simultaneously evaluated in the same individuals, particularly during acute dengue virus infection, remains unavailable. Consequently, it is not yet established whether the effects of *IL6* variation on cytokine production interact with the altered sIL-6R availability associated with rs2228145, or whether these two genetic axes independently contribute to inter-individual differences in IL-6 exposure and signaling.

This represents a major knowledge gap and an important limitation of the proposed IL-6 production IL-6R buffering model. The framework should therefore not be interpreted as demonstrating a genetic epistatic relationship between *IL6* and *IL6R* variants, but rather as providing a mechanistic hypothesis that can be experimentally tested. Future genotype-stratified studies should simultaneously assess relevant *IL6* and *IL6R* variants within the same dengue cohorts and integrate these genotypes with longitudinal measurements of IL-6, sIL-6R, sgp130, and downstream signaling and endothelial biomarkers. Such analyses could determine whether specific combinations of *IL6* and *IL6R* genotypes produce distinct IL-6 exposure or signaling phenotypes and whether these genotype combinations are associated with clinical severity. Experimental systems incorporating defined *IL6* and *IL6R* genotypes, including allele-specific genome editing, would further allow direct testing of whether altered IL-6 production and receptor buffering interact functionally to regulate systemic IL-6 homeostasis. Importantly, any such effects should be evaluated within the broader inflammatory network of dengue rather than interpreted as an isolated genetic determinant of disease pathogenesis. Therefore, to validate and advance this framework, future research must move beyond retrospective association studies and focus on mechanistically modeling these complex kinetic interactions. *In vitro* microfluidics, prospective clinical cohorts, precision clinical trials, endothelial shear stress models, kinetic multi-analyte assays, and genotype-guided enrollments all can be very useful strategies.

- *In Vitro* Microfluidics: Researchers should utilize organ-on-a-chip models to observe how varying ratios of IL-6, sIL-6R and sgp130 influence endothelial permeability under physiological shear stress.
- Prospective Clinical Cohorts: Large-scale prospective viral cohorts must implement serial, daily blood sampling to chart the precise mathematical kinetics of the free IL-6 index during the acute, critical, and recovery phases of infection.
- Precision Trial Design: Future clinical trials evaluating IL-6 inhibitors must reject unselected enrollment. Instead, trials should explicitly stratify patients based on their *IL6*/*IL6R* genotype and baseline soluble receptor buffering capacity.

## Conclusion

The unified IL-6-IL-6R framework provides a sophisticated, multi-layered model that reconciles host genetic variation with the complex realities of viral immunopathology. By demonstrating that acute inflammation is governed by a tightly coordinated system of cytokine production, buffering capacity, and receptor availability, this model moves the field past the limitations of traditional, production-centric analysis. Implementing this framework through targeted multi-analyte biomarkers and genotype-guided precision therapies holds immense promise. Ultimately, it allows clinicians to safely restore immune homeostasis, prevent severe endothelial dysfunction, and significantly improve patient outcomes during acute viral infections.
